# Imaging and liquid biopsy in the prediction and evaluation of response to PRRT in neuroendocrine tumors: implications for patient management

**DOI:** 10.1007/s00259-021-05359-3

**Published:** 2021-04-26

**Authors:** Wolfgang Roll, Matthias Weckesser, Robert Seifert, Lisa Bodei, Kambiz Rahbar

**Affiliations:** 1 Department of Nuclear Medicine, University Hospital Muenster, Albert-Schweitzer-Campus 1, 48149 Münster, Germany; 2 West German Cancer Center, Muenster and Essen, Essen, Germany; 3 Department of Nuclear Medicine, University Hospital Essen, Essen, Germany; 4 Department of Nuclear Medicine, Radiology, Memorial Sloan Kettering Cancer Center, New York, USA

**Keywords:** PRRT, Liquid biopsy, NET, SSTR-PET

## Abstract

**Purpose:**

The aim of this narrative review is to give an overview on current and emerging imaging methods and liquid biopsy for prediction and evaluation of response to PRRT. Current limitations and new perspectives, including artificial intelligence, are discussed.

**Methods:**

A literature review of PubMed/Medline was performed with representative keywords. The search included articles published online through August 31, 2020. All searches were restricted to English language manuscripts.

**Results:**

Peptide radio receptor therapy (PRRT) is a prospectively evaluated and approved therapy option in neuroendocrine tumors (NETs). Different ligands targeting the somatostatin receptor (SSTR) are used as theranostic pairs for imaging NET and for PRRT. Response assessment in prospective trials often relies on the morphological RECIST 1.1 criteria, based on lesion size in CT or MRI. The role of SSTR-PET and quantitative uptake parameters and volumetric data is still not defined. Monoanalyte tumor marker chromogranin A has a limited value for response assessment after PRRT. New emerging liquid biopsy techniques are offering prediction of response to PRRT and prognostic value.

**Conclusions:**

New response criteria for NET patients undergoing PRRT will comprise multiparametric hybrid imaging and blood-based multianalyte markers. This represents tumor biology and heterogeneity.

## Introduction

The incidence of neuroendocrine tumors (NETs) has increased over the years [1], with gastro-entero-pancreatic-NET being the most prevalent and the most aggressive of the NET-family [2]. Gastroenteropancreatic (GEP) NETs present with variable clinical behavior ranging from incidental discovery to hospitalization following hormone hypersecretion. Five-year survival rates range from 15 to 95% depending on the location of the primary tumor, differentiation and proliferation index, level of metastatic spread at diagnosis, and available treatments [3–6].

Management and therapy of these tumors are influenced by disease grade, stage, and underlying pathology. This includes surgical resection, drug therapy, and peptide radio receptor therapy (PRRT). As the majority of GEP-NET patients initially present with distant metastases, surgical cure is rare, and systemic therapies are needed [5, 7]. Following the results of prospective trials in the past 20 years, PRRT is increasingly used in metastatic, inoperable GEP-NET [8] and, as a result, has been approved by authorities as a second-line treatment after/in combination with somatostatin analogues (SSA) [9]. Patient selection is based on demonstration of adequate somatostatin receptor (SSTR) expression at scintigraphy or positron emission tomography (PET). These molecular imaging methods use radioactively labeled SSTR agonists with different affinities to SSTR-subtypes, as the PET-tracers [^68^Ga]Ga-DOTATATE, [^68^Ga]Ga-DOTATOC, and [^68^Ga]Ga-DOTANOC [10]. Cutoff levels of SSTR expression in ^68^Ga-SSTR-PET necessary for PRRT are not clearly defined; however, lesional uptake should exceed hepatic background, in order to achieve a satisfying radiation dose. This concept of semiquantitatively assessing uptake in the tumor was originally established by Krenning et al. for SSTR scintigraphy (^111^In-pentetreotide) [11] but is applied nowadays for SSTR-PET and post-therapy SSTR scintigraphy [12].

Response assessment is recommended 1–3 months after completion of four cycles of PRRT and includes morphological and/or functional PET imaging together with the assessment of clinical changes [9, 13], while Response Evaluation Criteria in Solid Tumors (RECIST) [14] for morphological imaging are well-established, their utility for NETs is questioned. In addition, standardized and validated response criteria for molecular imaging of NETs do not exist. The [^18^F]F-FDG-PET response criteria in solid tumors (PERCIST) are not directly transferrable to NET disease [15]. Although recently standardized criteria for SSTR-PET imaging were presented [16], criteria for response assessment with molecular SSTR-PET still present an unmet need [2, 5, 17].

In addition to the known limited accuracy in diagnosis and monitoring of NET disease, the default biomarker, chromogranin A (CgA), has little or no value in PRRT response evaluation. Its alterations are independent of the final treatment result [18, 19]. Recent innovative candidate biomarkers, such as the circulating multigenomic mRNA signatures, have already passed the early development stages and are being tested in diverse clinical scenarios, including PRRT [20].

In this review, we provide a comprehensive overview of different imaging and blood biomarkers that are used for the prediction and the evaluation of response to PRRT in GEP-NET. We anticipate current implications for patient care and future perspectives that might improve management of GEP-NET patients before and after PRRT.

### Review criteria

A literature review of PubMed/Medline was performed with keywords, including “NET,” “neuroendocrine tumor,” “PRRT,” “response assessment,” “liquid biopsy,” “biomarker,” “MRI,” “PET,” and “scintigraphy.” The search included articles published online through August 31, 2020. All searches were restricted to English language manuscripts. After excluding publications that primarily focused on PRRT in NET, the remaining publications were reviewed and discussed in the article if suitable.

## Imaging

Imaging of NET plays a fundamental role for staging, treatment selection, response assessment, and follow-up. Techniques used include morphological imaging methods, such as computed tomography (CT) and magnetic resonance imaging (MRI). Moreover molecular imaging methods are used, such as scintigraphy with ^111^In or ^99m^Tc labeled SSA, or PET with [^68^Ga]Ga -labeled SSA or other PET-tracers ([^18^F]F-FDG). Previously widely used scintigraphy with [^111^In]In or [^99m^Tc]Tc-labeled SSA is more and more replaced by SSTR-PET. SSTR scintigraphy underestimates SSTR expression in small NET metastases [21] and is not predictive of progression-free survival [8]. Sensitivity and specificity of imaging can be increased by combining morphological and functional imaging, which might also optimize response assessment. As the most common site of distant metastases of NET is the liver and the presence of hepatic metastases constitutes an important factor for survival [22], many studies on morphological response assessment focus on liver metastases.

## Morphologic imaging

### CT

NET metastases, especially of the liver and primary tumors, are often hypervascularized with rich contrast enhancement (CE) in the early arterial phase. Assessment of CE by measuring arterial tumor attenuation based on Hounsfield units might yield complementary information to evaluation by RECIST 1.1. Furthermore, in a retrospective analysis, the fold change of arterial tumor attenuation before and after PRRT correlated with progression-free survival (PFS) [23]. However, in this study, only one liver metastasis with the highest Hounsfield unit was assessed, not taking into account tumor heterogeneity in metastatic disease.

### MRI

Overall, CE MRI has a higher sensitivity than CE-CT in detecting NET metastases and allows for a more accurate detection of liver metastases compared to CT and ultrasound [24]. In conventional MRI, T1-weighted hepatic arterial phase and T2-weighted fast spin echo sequences are primarily used. Additional liver-specific MR contrast agent and diffusion-weighted imaging (DWI) might even improve sensitivity [24, 25]. DWI allows to quantify the Brownian motion of water molecules, indirectly reflecting the cellularity of a tumor by the apparent diffusion coefficient (ADC). Additional parameters related to intravoxel incoherent motion (IVIM), as diffusion (*D*), perfusion fraction (*f*), and pseudo-diffusion (*D*∗), might also be assessed. DWI is used for response assessment in various tumor entities, such as prostate cancer [26] or hepatocellular-carcinoma [27], as well as in liver metastases of NET after local therapies [28]. Preliminary results on DWI for response assessment of patients undergoing PRRT present with heterogeneous results in small patient cohorts. Weber et al. could not find DWI parameters that were statistically significantly different between responders and non-responders to PRRT, and thus, no conclusion regarding prediction of response could be drawn [29]. Weikert et al. provide data that ADC values might differentiate regressive from progressive liver metastases in MRI as early as 48 h after PRRT, whereas the IVIM parameter did not show a significant correlation [30].

Dynamic contrast-enhanced (DCE) MR imaging has already proven useful for the assessment of therapy induced changes in other tumors [31]. In preclinical evaluation, DCE and other MR-derived biomarkers assessed tumor tissue response after PRRT [32]. Data on the use of DCE for the assessment of response in patients undergoing PRRT is still sparse. Compared to DWI and post-therapy scintigraphy, DCE-parameter did not show additional benefit in early response assessment 48 h after PRRT [30].

### Criteria for response assessment

Response assessment by morphological imaging is widely accepted. It is used to assess response after PRRT in retrospective analysis [33] and to assess primary end point PFS in large prospective studies as in the NETTER-1 trial [8]. RECIST, updated in 2009 to RECIST 1.1, is based on the evaluation of cross-sectional images of CT and MRI. Target lesions are defined in pretherapeutic baseline scans, measured and subsequently monitored in follow-up scans [14, 34]. Thus, RECIST allows for a standardized metric evaluation of lesion diameter and a good level of interobserver reliability. However, the heterogeneity and composition of the lesion as well as its viability are not considered [35]. This results in general limitations including the differentiation of disease stabilization and pseudo-progression and a lack of sensitivity in the determination of progressive disease [5]. Examples of response to therapy not adequately captured by RECIST criteria are, for example, change in composition of prognostically relevant liver metastases. As a response to PRRT, the tumor tissue in these metastases is replaced by necrosis and hyperperfusion in arterial phase is decreasing, while the diameter remains unchanged. Especially, low-grade NETs exhibit slow growth, being difficult to assess by morphological imaging. Moreover RECIST 1.1 only considers a maximum of five tumor lesions and a maximum of two lesions per organ [34]. This results in an inadequate assessment of metastatic disease. This is of particular relevance in NET, as the majority of patients with GEP-NET have distant metastasis at diagnosis [2, 7]. Another concern is that bone metastases are often non-target lesions in RECIST 1.1 and are generally difficult to assess in morphological imaging [5]. Increasing calcification of bone metastases may be observed in case of progression, but as well as in NET metastases responding to therapy. Response according to RECIST 1.1 is defined as tumor shrinkage. However, PRRT in GEP-NET patients often results in disease stabilization, challenging the suitability of RECIST 1.1 in GEP-NET response assessment [5, 18]. As a better way of reflecting this tendency to exhibit slower growth, Choi criteria, originally developed for slowly growing gastrointestinal stroma tumors, were also applied in the evaluation of treatment response in NET [36, 37]. Using RECIST 1.1 and Choi criteria, Huizing et al. could show in a retrospective analysis that progressive disease in morphological imaging is indicative of poor survival [33].

## Molecular imaging

### SSTR-PET

Imaging SSTR by positron emission tomography is realized by the combination of a positron emitter, 68Ga, a chelator, DOTA, and an SSA. Different SSA, −TOC, −NOC, and −TATE, show different affinities to SSTR-subtypes. However, they all bind to SSTR2, the receptor subtype predominantly expressed on GEP-NETs [38]. Recently, radiopeptides with antagonist effects on SSTR have been introduced. First results indicate an increased sensitivity, especially for liver metastases [39]. However, because of limited data and clinical experience especially concerning response assessment around PRRT only studies including SSTR-PET using [^68^Ga]Ga -labeled DOTA-agonists are discussed.

Elevated pretherapeutic SSTR expression quantified by [^68^Ga]Ga-DOTA-SSA PET is a predictor for the outcome of PRRT and might thus also have an impact on PFS [40, 41]. Volumetric assessment of all PET-positive lesions before radioligand therapy has already proven impact on overall survival (OS) in prostate cancer patients undergoing prostate-specific membrane antigen (PSMA) therapy [42]. A first study has shown similar results for the SSTR-positive tumor volume in [^68^Ga]Ga-DOTATATE PET at initial diagnosis as being predictive of PFS (threshold: 50% of local standard uptake value (SUV)max) [43]. There are no studies investigating the role of SSTR-positive tumor burden before PRRT in NET.

The inadequate assessment of tumor heterogeneity is a well-described shortcoming of standardized morphological and functional response assessment. First results indicate that quantitative [44, 45] and visual [46] assessment of tumor heterogeneity has a predictive and prognostic value in NET patients undergoing PRRT.

Interpretation of change in quantitative uptake values in SSTR-PET during and after PRRT is challenging. Reduced binding of SSTR agonists can indicate a reduced number of SSTR either due to disease progression, a therapeutic effect, or other parameters such as changes in perfusion or dedifferentiation. Thus, the change in tumor uptake in SSTR-PET adapted by PERCIST or European Organization for Research and Treatment of Cancer (EORTC) criteria has not yet convincingly been shown to reflect response to PRRT [29, 47] or to predict OS after PRRT in NET [33]. All these studies focus on the assessment of uptake values in few, often only one or two target lesions. This results in the identical disadvantage as assigned to morphological criteria, not capturing the common clinical situation of widespread metastatic disease. Moreover, one individual might present with different quantitative uptake values in longitudinal SSTR-PET-scans, due to different scan conditions, including different exposure to cold SSA (OFF vs ON) [48]. Measuring relative uptake values compared to reference tissue might partly overcome this limitation [49]. However, a promising new way, also adapted from above mentioned criteria, might be to quantify SSTR-positive tumor volume [43, 50, 51] or tumor heterogeneity [44]. These parameters were not yet used for response assessment after PRRT in GEP-NET.

A well-described advantage of early or late response assessment of SSTR-PET after PRRT is the early detection of new distant metastases resulting in predictive value for early diagnosis of therapy failure (unequivocal progression) [2, 33, 47].

### [^18^F]F-FDG

[^18^F]F-FDG-PET targets increased glycolytic metabolism and is generally recommended for imaging of NETs with high proliferation index (G3) and poorly differentiated neuroendocrine carcinomas. While per patient positivity may account for around 60% of G1 and G2 NETs, per lesion positivity is lower, due to disease heterogeneity. These values are typically not provided in the majority of studies. As a consequence, [18F]F-FDG-PET is not part of the routine clinical workup in well-differentiated tumors [52]. However, with increasing Ki-67, SSTR-PET becomes less reliable and additional [^18^F]F-FDG-PET should be considered.

**Fig. 1:**
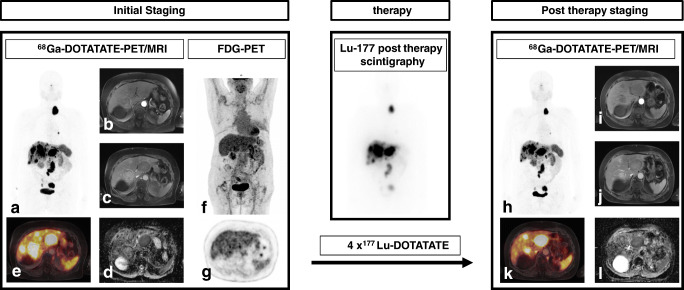
An 80-year-old patient with liver and lymph node metastases of NET (G2; Ki-67: 19%) with unknown primary. Maximum intensity projection (MIP) of 68Ga-DOTATATE PET (**a**) shoes high level of SSTR expression, higher than uptake in liver and spleen, an advantage for PRRT. MRI (**b**, **c**) shows typical early arterial hyperenhancement (**b**) and low ADC values (**d**), consistent with malignant lesions, as shown in fused images (**e**). [18F]F-FDG-PET MIP (**f**) and transversal PET of the liver (**g**) did not show elevated uptake in NET metastases, being a favorable prognostic marker in highly proliferative NET. 177Lu-post-therapy whole-body scan after the first of four cycles of 177Lu-DOTATATE therapy proved high uptake metastases of NET (Krenning scale 4; higher than uptake in liver and spleen). Post-therapy staging by 68Ga-DOTATATE PET/MRI (**h**) revealed mixed response with partly constant (seg. II) and partly regressive liver metastasis (seg. V) (**i**, **j**) with continuously low ADC values (**l**). A situation difficult to assess by current response assessment criteria. Furthermore, fused images (**k**) and MIP (**h**) show reduced SSTR expression and reduced SSTR-positive volume compared to initial staging. As these parameters are not integrated into current response assessment criteria, its role for outcome prediction remains unclear

Increased uptake in ^18^F-FDG-PET provides parameters which are predictor of response to PRRT and prognosticator of survival in NET patients in mixed collectives of G1, G2, and G3 NETs [53–55]. In well-differentiated G1 and G2 NETs, negative [18F]F-FDG-PET scans were linked to a significantly better PFS after PRRT regardless of the Ki67 grading score [52, 56]. A major confounder when linking [18F]F-FDG-positivity to response to treatment is that increased uptake in [18F]F-FDG-PET is a prognostic factor for survival regardless of the treatment applied [55, 57]. In combination with SSTR-PET, [18F]F-FDG-PET allows for the assessment of tumor heterogeneity in patients with metastases (Fig. 1). In contrast to the evaluation of Ki-67 in a biopsy specimen of a selected lesion, PET offers a whole-body evaluation. Moreover, Ki-67 assessment, often at initial diagnosis, does not necessarily reflect the tumor grading at the imaging time point, in general months to years later.

### Criteria for response assessment

Molecular functional imaging of SSTR expression is not yet fully integrated into response criteria for NET-treatment [2] (Fig. 1). Thus, well-established criteria for [18F]F-FDG-PET imaging, PERCIST and EORTC criteria, are often adapted, without having been validated for response assessment with SSTR-PET [33, 51]. Criteria are based on the change in SUV and volumetry might also be applied. The volumetric approach uses thresholding based on uptake in reference tissue (e.g., liver or blood pool) [15]. Whereas PERCIST criteria for [18F]F-FDG-PET focus on SUVpeak, the SUV in a 1-cm^3^ spherical volume of interest (VOI), EORTC criteria are based on SUVmax, the maximum SUV in a single voxel [15, 58]. The major drawback of assessing the change in SSTR expression is its interpretation, e.g., of decrease of these parameters, either as response to therapy or dedifferentiation of the tumor often resulting in disease progression (Table 1). Beyond quantification in certain voxels and VOIs, PERCIST criteria and international guidelines on [18F]F-FDG-PET implicate volumetric assessment of PET-positive lesions, to assess whole tumor burden. An increasing number of studies are transposing volumetric evaluations adapted from [18F]F-FDG-PET by using different methods of thresholding (percentage of local SUVmax, calculated from uptake in reference tissue). These studies focus on SSTR-PET in GEP-NET at an earlier time point of disease or on response assessment of PRRT in other tumors [43, 50, 65]. Neither of these methods have been validated in prospective trials evaluating the outcome according to the change in uptake. Further advances to overcome limitations of current criteria are preliminary studies on the predictive and prognostic value of assessment of lesion heterogeneity before PRRT [44, 45]. This approach is a promising future tool also for response assessment after PRRT.

**Table 1:** Promises and drawbacks of imaging and liquid biopsy–based approaches for prediction and evaluation of response after PRRT

	Pro	Contra
CT	- Fold change in arterial tumor attenuation might yield complementary information to RECIST 1.1. [23].	- Inferior to MRI for the assessment of liver metastases [24]
MRI	- Superior to other morphological imaging modalities, especially fort the assessment of prognostically relevant liver metastases [24, 25] - Early change in ADC_mean_ values might differentiate regressive from progressive liver metastases after PRRT [30]. - DCE-MRI has been useful in preclinical evaluation of different MR-derived biomarkers for tumor tissue response after PRRT [32].	- DWI parameters did not predict or correctly assess response to PRRT [29]. - Early changes in IVIM and DCE-parameters (48 h p.i.) were not significantly different between responders and non-responders to PRRT [30].
SSTR-PET	- Pretherapeutic SSTR expression (baseline SUV_max_) predictor for the PRRT outcome [40, 41] - SSTR-positive tumor volume (threshold 50% of SUVmax) has prognostic value (PFS) [43]. - Improved early detection of new distant metastases compared to morphological imaging [2, 33, 47] - Early per-cycle reduction of SUV tumor to spleen ratio independent predictor of TTP after PRRT [49] - Tumor heterogeneity at pre-PRRT SSTR-PET predicts survival (textural features, entropy vs. PFS and OS [44, 45], visual assessment vs. TTP after PRRT and OS [46]).	- 68Ga-DOTA-TOC PET shows no advantage over conventional anatomic imaging for assessing response to PRRT (reading [47] and change in SUVmax and SUVmean [29]). - No studies on SSTR-positive tumor volume around PRRT - Pretherapeutic SSTR expression did not correlate with PFS or OS after PRRT (SUVmean [44, 45], SUVmax [43–45]). - Change in SUVmax and SUVmean did not predict overall survival after PRRT [33]. - AI not yet applied for the assessment of tumor heterogeneity in SSTR-PET around PRRT.
FDG-PET	- Pretherapeutic [18F]F-FDG-PET provides parameters which are predictive of response to PRRT (SUVmax [54], SUV 2.5 as cutoff for grading PET-positive/negative [52]). - Uptake in [18F]F-FDG-PET provides parameters which are prognosticator of survival (OS or PFS) in NET patients (SUVR (lesion SUV_max_ to SUV_mean_ liver) [53], discordant FDG and somatostatin receptor uptake [55], SUV 2.5 as cutoff for grading PET-positive/negative [56]).	- Increased uptake in [18F]F-FDG is a prognosticator of survival in NET patients regardless of the treatment applied (SUVmax [57], discordant [18F]F-FDG, and somatostatin receptor uptake [55]).
Post-therapy Scintigraphy	- Krenning scale, in post-therapy scintigraphy, was significantly higher in lesions, responding to PRRT compared to stable or progressive lesions [30]. - Tumor doses (dosimetry) correlated with reduction of tumor size [59]. - New lesions are indicative of therapy failure.	- No/limited data in response assessment or prediction of outcome - Limited spatial resolution compared to SSTR-PET
Monoanalyte markers	- Baseline CgA concentration of greater than 600 ng/mL is an independent risk factor of shorter PFS after PRRT [60].	- Inferiority of CgA for outcome prediction (PFS or OS) in comparison to textural imaging parameter (entropy) [44, 45] and FDG-PET parameters (SUV_max_) [57] - CgA could not predict PFS after PRRT in prospective NETTER I trial [8].
Multi-analyte markers	- NETest: multigene expression–based assay correlating 51 circulating mRNA (NETest) assesses response to PRRT with high accuracy [61] - PPQ: predicts response to PRRT (correlation to RECIST 1.1 and NETest [61, 62]) - PPQ: is a highly specific predictor of the efficacy of PRRT [63]	- ctDNA: No/limited data for response prediction to PRRT for ctDNA-based approaches, detection of specific mutations, identification of chromosomal and transcriptional alterations [64]

Recent advances in structural reporting of SSTR-PET by SSTR-RADS 1.0 [66] offer high interobserver reliability [67] necessary for reliable response assessment. These might pave the way for structured response assessment criteria including SSTR-PET.

Response assessment by molecular functional imaging was first established by Krenning et al. for uptake evaluation of the tumor compared to uptake in reference organs liver and spleen in whole-body SSTR scintigraphy ([^111^In]In-pentretreotide; Octreoscan) [11]. These criteria were adapted for response assessment in post-therapy [^177^Lu]Lu-scintigraphy after PRRT in GEP-NET patients as well as in other disease treated with PRRT [30, 65]. Its use is widely accepted [8].

### Post-therapy scintigraphy

After PRRT with beta-emitting [^177^Lu]Lu-labeled-DOTA-SSA, its gamma component allows to image and to quantify tumor uptake. Quantification of Bremsstrahlung images, necessary for post-therapy scans of [^90^Y]Y-labeled-DOTA-SSA because of the lack of y-emission, is rather difficult [68]. Tumor doses, ranging from 10 to 340 Gy by post-therapy dosimetry, are correlated with reduction of tumor size in 2.2–4.0-cm tumors (*r* = 0.64) and showed a stronger correlation for tumors larger than 4.0 cm (*r* = 0.91) [59]. The role of post-therapy scintigraphy for response assessment is currently unclear (Fig. 1). The lesion to spleen uptake ratio, adapted from Krenning scale, was significantly higher in lesions, responding to PRRT compared to stable or progressive lesions [30]. A critical shortcoming of this approach is that reduced signal (decreasing number of SSTR) might be associated with radiation-induced cellular death, albeit SSTR expression might also evolve during disease course. Similar to post-treatment SSTR-PET, new lesions are indicative of therapy failure.

## Liquid biopsy

### Standard biomarkers

CgA and NSE have been historically considered circulating tumor markers of NET. CgA can be a surrogate of tumor burden, which has prognostic impact. Whereas in one study on PRRT a baseline CgA concentration of greater than 600 ng/mL was a risk factor for early progression [60], other studies report on the inferiority of CgA for outcome prediction in comparison to textural imaging parameter [44]. Similar results were described by the same group for NSE elevations, for a correlation with OS [69]. However, in the NETTER-1 trial, as well as in other retrospective analyses, circulating biomarkers such as CgA, NSE, and 5-hydroxyindole-3-acetic acid could not predict response to PRRT or survival [8, 33, 49]. In conclusion, these blood parameters may indicate prognosis instead of PRRT-responsiveness [70]. The major concerns are the low sensitivity and specificity, including false-negative results in the non-secretory tumors and false-positive results related to proton pump inhibitor use, kidney failure, and heart disease [5, 71]. Insulin, gastrin, and vasoactive peptide (VIP) allow for highly accurate measurement of tumor secretory activity. However, these tumors comprise only a very small group of NETs and are partly not treatable with PRRT due to limited SSTR expression. In the majority of disease, evaluation of monoanalyte secretory products fails to represent the multiplicity of malignancies and does not correlate with morphological response assessment [72, 73].

### New biomarkers

Recently, quantification of multi-analyte tumor (associated) products has gained substantial interest for their capacity to assess tumor heterogeneity. These comprise circulating tumor DNA (ctDNA), circulating tumor cells (CTC), and mRNA. Whereas in other tumors analysis of ctDNA, CTC, or methylated gene targets has shown promising results [74], its clinical utility in NET is limited [75]. NETs are mutationally relatively quiet tumors. In contrast to other tumors, activating mutations are rare in NET. Albeit, somatic mutations in tumor suppressor genes, e.g., multiple endocrine neoplasia (MEN) 1, occur fairly frequently. Next-generation sequencing approaches testing ctDNA try to identify potential therapeutic targets in the genomic landscape of NET [48]. However, the clinical usefulness of this approach and other alterations, such as *ATRX*, *DAXX* [76], or *YY1* [77], as well as alterations in copy number and chromosomal imbalances remains to be proven [20]. Currently, these ctDNA-based approaches, including next-generation sequencing, are not used in combination with PRRT. They have thus no impact on prediction of response or outcome. Current research related to PRRT in NET focuses on mRNA-based approaches for liquid biopsy.

### NETest

**Fig. 2:**
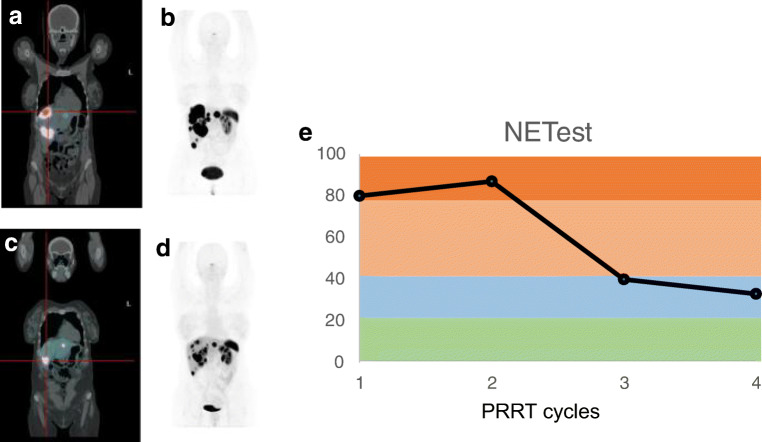
Utility of the NETest and PPQ for stratification and monitoring of PRRT in a patient affected by a well-differentiated G2 neuroendocrine tumor of unknown primary metastatic to the liver, status post somatostatin analogues. The patient is selected for PRRT with a positive PPQ (predicted to respond) and receives ^177^Lu-DOTATATE (26 GBq in 4 cycles). The pretreatment imaging (**a**, **b**, fused and pure ^68^Ga-DOTATATE images) demonstrates bilobar liver metastases. The restaging exam performed 5 months later (**c**, **d**, fused and pure SSR PET images) demonstrates a decrease in size and avidity of the liver metastases, thus confirming the PPQ prediction. The NETest levels (**e**, activity 1–100%, positive >20) measured throughout the therapy are decreased during the treatment cycles

The NETest is a multigene expression–based assay correlating 51 circulating mRNA specific to NET, and providing a molecular biological characterization of tumoral behavior [2] (Fig. 2). This test outperforms single-analyte measurements for diagnosis [78] and therapy response assessment and allows for correlation to clinical status [79]. In contrast to CgA, high NETest levels could separate progression from stability, at the time of the analysis. Moreover, NETest indicates relapse or progression of disease months in advance compared to imaging [80–82].

In assessing treatment response to SSA, NETest was far more accurate than CgA. NETest elevations occurred at an earlier time point in a prospective blinded study, while CgA was noncontributory. In contrast to CgA, this marker was able to predict response to SSA treatment [81]. NETest also accurately monitors response to PRRT (accuracy 98% to gold standard RECIST 1.1), resulting in a decrease of NETest values in RECIST 1.1 responders to PRRT. This makes NETest an effective surrogate marker of interim radiological response according to RECIST 1.1 obtained *after* PRRT. Moreover, follow-up NETest categories stable vs progressive significantly correlated with median progression-free survival [61].

### PPQ

For the prediction of response to PRRT, the PRRT predictive quotient (PPQ) was developed using specific subsets of circulating transcripts coupled with tumor grading [62]. Pattern of gene expression included in PPQ reflect genes involved in growth factor signaling and metabolism. Elevated levels of gene expression were associated with an area under the curve of 0.74 for prediction of absence of progression on PRRT. As already shown before, patients with low and intermediate grade NET or carcinoids were more likely to present with response or stable disease in the same study. Logistic regression modeling integrating gene expression and grading resulted in a predictive quotient with high accuracy (AUC: 0.9). The output of the PPQ is either a positive (predicts response to therapy) or a negative (non-responders) prediction of response to PRRT. The predictive accuracy of this output was 94% for response to PRRT [62]. Moreover, pretreatment prediction by PPQ correlated not only with RECIST 1.1 (accuracy 97%) response assessment but also with response assessment by NETest (accuracy >97%) [61].

In a prospective validation in three European centers, median progression-free survival was not reached in the group of patients classified at baseline as PPQ-positive. Median progression-free survival was 10–14 months for PPQ-negative patients (from start of treatment; *p* < 0.0001). Sensitivity was 94–97%, and the negative predictive value 83–93% [63]. In this study, the three prospective validation cohorts were examined to provide data on the specific predictive value of PPQ, namely specific for PRRT. In contrast to this therapy-associated specific predictive value, other biomarkers, such as [18F]F-FDG positivity and elevated CgA, are prognostic. They indicate a worse outcome regardless of the treatment applied. The predictive value, hence, the specificity of the PPQ for PRRT, was tested in two additional cohorts, one receiving SSA treatment, the other undergoing watchful waiting. PPQ could not predict the outcome (PFS) of either of these non-PRRT groups [63]. Thus, PPQ is a specific biomarker for the prediction of response to PRRT and represents a radiation sensitivity fingerprint in blood [64].

## Future perspectives

### Hybrid imaging

Studies from previous decades were partly only looking at PET alone compared to CT [47]. State-of-the-art response assessment should include all available molecular and morphological information of the individual tumor. SSTR-PET-CT/PET-MRI overcomes limitations of previously used SSTR scintigraphy and is the new standard of care in NET patients. SSTR-PET-MRI [29], including modern MR techniques as DWI or DCE-MRI, will be increasingly important for response assessment in NET. Preliminary studies of response assessment after PRRT already included advanced MR features [30]. These two techniques are especially important in the assessment of liver metastases, which are a prognostic factor of survival in well-differentiated NETs. However, the clinical impact of combined PET-MRI versus serial PET-CT and MRI is still under discussion [83]. In clinical routine, functional imaging in NET is currently limited to identification of membrane receptors and assessment of glucose utilization. The use of different radiopharmaceuticals to assess proliferative or other molecular tumor properties and its combination with blood-based molecular information will provide a deeper insight into individual NET biology in real time. Especially hybrid imaging with its whole-body imaging approach enables assessment of tumor heterogeneity at every imaging time point for every lesion. The use of state-of-the-art morphological imaging and multi-tracer approaches might enhance our understanding of the tumor without the need for repetitive invasive procedures.

### Artificial intelligence

Artificial intelligence (AI) offers new opportunities for response assessment and prediction of survival. This includes the extraction of more data, beyond information acquired in routine clinical workup, as size, contrast enhancement, and quantitative uptake values. The use of sophisticated algorithms allows for automated post- processing of images acquired in clinical routine. Thus, this approach might overcome conventional image analysis with manual feature extraction, although these “conventional” parameters have already provided promising results for outcome prediction after PRRT [44, 46]. First results were reported on the application of AI to hybrid [18F]F-FDG-PET-CT/MRI images, e.g., in lung cancer [84] or brain tumors [85]. Moreover, AI might improve not only tumor assessment but also technical aspects of hybrid imaging, e.g., dose reduction [86] or attenuation correction [87].

The biggest advantage is that artificial intelligence can not only be applied to morphological imaging and [18F]F-FDG-PET but also applied to SSTR-PET, albeit data is still sparse. The aspect of whole-body SSTR-PET protocols facilitates fast, automatic, whole-body tumor burden assessment. By manual segmentation, whole-body tumor burden assessed by PET can already be correlated to survival [43]. PET-MRI offers data on whole-body SSTR status and high-end morphological data, especially for the frequent and highly prognostic hepatic metastases. The assessment of tumor heterogeneity by manual or semiautomatic measurement of SUV has prognostic value in NET [55]. AI has the potential to assess complex multiparametric morphological and functional imaging data, offering a fingerprint of the underlying pathology and the frequency of alterations in different tissue parameters.

Before reaching its full potential, wide application of AI requires standardized examinations. Training of sophisticated algorithms on large prospective trials with high-quality data, including relevant outcome information, is mandatory.

### Novel criteria of response assessment

Although SSTR-PET is used in everyday practice as a gatekeeper for patients before undergoing PRRT, data of this imaging method is not incorporated into frequently used response assessment criteria. RECIST 1.1 has well-described shortcomings, as only morphological information is included. First, structured PET response criteria PERCIST, however, are only available for [18F]F-FDG-PET. This underlines the need for response assessment criteria for SSTR-PET and other incoming theranostics. Recent advances in structured reporting systems for SSTR-PET might help to overcome current use of data from SSTR-PET in an empirical, non-standardized way. As results for the predictive and prognostic significance of quantitative uptake values from SSTR-PET are inconclusive, new parameters assessing tumor volume and heterogeneity should be included. Data on the predictive or prognostic role of quantitative SSTR-PET uptake values are in the majority of cases extracted from retrospective data sets. Instead, we need a fundamental understanding of change of conventional PET-derived parameters under therapy that can be generated from large prospective trials or by data-mining, including high-quality data from large centers. This pooling of retrospective data sets is currently hampered by the limited standardization of PRRT and SSTR-PET, including the use of different radioligands for imaging and therapy. To ensure repeatability of PET measurements, currently associated with relevant variability [88], harmonization of imaging procedures is required. Recent efforts for [18F]F-FDG-PET harmonization [89, 90] need to be applied to SSTR-PET, although many sources of error can be overcome by complying with international guidelines [10, 48]. The current variability of SSTR-PET measurements underlines the need for generally applicable response assessment criteria. These new criteria should then be (re-)evaluated on large prospective datasets, as performed for PERCIST criteria [88].

### Implementation into clinical practice

Capturing the overall clinical status of the patient by providing an insight into molecular biology of the tumor, multi-analyte liquid biopsy overcomes certain disadvantages of tissue-based analysis. Hence, blood-based information remains the new frontier of longitudinal patient management offering real-time information. Multi-analyte biopsy might be included into routine clinical-based and imaging-based assessment before PRRT. Assessing the molecular biology and heterogeneity of the tumor by a multiparametric approach including all the aforementioned imaging and liquid biopsy options will improve response assessment in NET patients [17]. Together with prospectively evaluated, standardized hybrid imaging-derived metrics, predictions on the possible effectiveness of PRRT might be possible in the future. New developments of multi-analyte biopsy-based prediction of response to other therapies than PRRT are warranted. These might be used to guide clinical decision-making towards other therapeutic alternatives in patients not suitable for PRRT. During and early after PRRT monoanalyte marker CgA is of limited value for response assessment. Thus, multi-analyte response assessment might offer advances in predicting and monitoring treatment efficacy. PRRT standard protocols typically consist of four applications over 6–8 months with post-therapy evaluation 3 months after the last cycle, resulting in substantial costs for health care systems [63]. Approximately 15–30% of patients will progress during PRRT. These patients would benefit from early response assessment or response prediction in terms of therapy adjustment, including combinations or alternatives [8, 61, 63, 91]. Apart from the benefit for the patient, this might be a health economic cost argument. NETest assesses therapy response with high accuracy helping to reduce the costs of imaging and of non-efficacious use of SSA [5, 80].

New prospectively evaluated criteria for response assessment based on hybrid imaging should overcome previously described shortcomings. Imaging-based criteria, associated with additional predictive and prognostic value, are warranted to complement blood-based assessment.

#### Conclusion

Strategies for modern treatment in oncology are no longer only based on tissue biopsy followed by tumor marker measurements and assessment of tumor size. In the era of precision imaging and liquid biopsy, capturing the complexity of tumor biology in real time will become standard of care. Hybrid imaging, including modern SSTR-PET, will be included into response assessment criteria. AI offers new opportunities for the assessment of tumor heterogeneity from hybrid imaging data acquired in routine clinical workup. Classical monoanalyte tumor markers, such as CgA, have been demonstrated to be inadequate to assess the treatment response. These markers are surpassed by the blood-based multigene biomarker NETest, which demonstrated 98% accuracy for early PRRT monitoring. In addition, the multigene test PPQ demonstrated to be an accurate specific prognosticator for PRRT response. Future strategies for response assessment and outcome prediction of PRRT will comprise the integration of state-of-the-art hybrid imaging of current and new theranostics with blood-based molecular information capturing the complexity of metastatic NET in the individual patient.

## Data Availability

Not applicable.
